# Adjunctive Rifampin Therapy For Diabetic Foot Osteomyelitis in the Veterans Health Administration

**DOI:** 10.1001/jamanetworkopen.2019.16003

**Published:** 2019-11-22

**Authors:** Brigid M. Wilson, Mary T. Bessesen, Gheorghe Doros, Sheldon T. Brown, Elie Saade, John Hermos, Federico Perez, Marion Skalweit, Brad Spellberg, Robert A. Bonomo

**Affiliations:** 1 Research Service and Geriatric Research, Education, and Clinical Center (GRECC),VA Northeast Ohio Healthcare System, Cleveland; 2 Division of Infectious Diseases, Case Western Reserve University School of Medicine, Cleveland, Ohio; 3 Division of Infectious Diseases, University of Colorado, Denver, School of Medicine, Department of Veterans Affairs Eastern Colorado Healthcare System, Aurora; 4 Massachusetts Veterans Epidemiology Research and Information Center, Boston; 5 Boston University School of Medicine, Boston, Massachusetts; 6 James J. Peters Veterans Affairs Medical Center, Bronx, New York; 7 Icahn School of Medicine at Mount Sinai, New York, New York; 8 Research Service, VA Northeast Ohio Healthcare System, Cleveland; 9 University Hospitals of Cleveland, Cleveland, Ohio; 10 Veterans Affairs Boston Healthcare System, Boston, Massachusetts; 11 Department of General Internal Medicine, Boston University, Boston, Massachusetts; 12 Medicine Service and GRECC, VA Northeast Ohio Healthcare System, Cleveland; 13 Department of Medicine, Case Western Reserve University School of Medicine, Cleveland, Ohio; 14 Department of Biochemistry, Case Western Reserve University School of Medicine, Cleveland, Ohio; 15 Los Angeles County and University of Southern California Medical Center, Los Angeles; 16 Department of Medicine, Keck School of Medicine of University of Southern California, Los Angeles; 17 Department of Pharmacology, Case Western Reserve University School of Medicine, Cleveland, Ohio; 18 Department of Molecular Biology and Microbiology, Case Western Reserve University School of Medicine, Cleveland, Ohio; 19 Department of Proteomics and Bioinformatics, Case Western Reserve University School of Medicine, Cleveland, Ohio; 20 CWRU (Case Western Reserve University)–Cleveland VAMC Center for Antimicrobial Resistance and Epidemiology, Cleveland, Ohio

## Abstract

**Importance:**

Among patients diagnosed with diabetes, the lifetime incidence of foot ulcers is 15%. Infection is a common complication of foot ulcers, and 20% to 60% of infections result in diabetic foot osteomyelitis (DFO). Current treatment guidelines do not endorse any specific antibiotic agent for DFO, but small clinical trials suggest the addition of rifampin to antimicrobial regimens results in improved cure rates for osteomyelitis.

**Objective:**

To compare the clinical outcomes of patients treated for DFO in the Veterans Health Administration (VHA) with and without adjunctive rifampin.

**Design, Setting, and Participants:**

This observational cohort study used VHA databases to identify index DFO cases from January 1, 2009, through December 31, 2013, and analyzed patients alive and without high-level amputation at 90 days after diagnosis in whom antibiotic therapy was initiated within 6 weeks of diagnosis. Patients with death or major amputation within 90 days of diagnosis, who were not treated with systemic antibiotics dispensed by the VHA within 6 weeks of diagnosis, or who were treated at facilities where rifampin was not dispensed for DFO were excluded. The retrospective cohort to inform the planning of a multisite randomized clinical trial was first investigated in spring 2015; retrospective analysis was performed from February 2017 through September 2019.

**Exposures:**

Patients initiating rifampin therapy within 6 weeks of the DFO diagnosis and receiving the drug for at least 14 days within 90 days of diagnosis were considered treated with rifampin. Patients not administered rifampin within 90 days of diagnosis served as the comparator group.

**Main Outcomes and Measures:**

A combined end point of mortality or amputation within 2 years of diagnosis was analyzed. Differences in times to event were evaluated using log-rank tests. Differences in event rates were compared using χ^2^ tests and multivariable logistic regression.

**Results:**

The analysis population included 130 patients treated with rifampin and 6044 treated without rifampin (total of 6174; 6085 men [98.6%]; mean [SD] age, 64.9 [9.7] years). Lower event rates were observed among the rifampin group (35 of 130 [26.9%] vs 2250 of 6044 [37.2%]; *P* = .02). Patients treated with rifampin were younger (mean [SD] age, 62.2 [9.4] vs 64.9 [9.6] years), had fewer comorbidities (mean [SD] Charlson comorbidity index score, 3.5 [1.8] vs 4.0 [2.2]), had more infectious disease specialty consultations (63 of 130 [48.5%] vs 1960 of 6044 [32.4%]), and more often had *Staphylococcus aureus* identified in cultures (55 of 130 [42.3%] vs 1755 of 6044 [29.0%]) than patients not treated with rifampin. A logistic regression estimating the odds of events and controlling for these and other covariates yielded a significant association of rifampin (odds ratio, 0.65; 95% CI, 0.43-0.96; *P* = .04).

**Conclusions and Relevance:**

In this cohort study, patients administered rifampin experienced lower rates of death and amputation than patients not treated with rifampin, which remained significant after adjustment for confounders. These results coupled with existing evidence from small clinical trials suggest the addition of rifampin to current treatment regimens may be a useful antimicrobial option in the treatment of DFO.

## Key Points

**Question:**

What are the amputation and mortality outcomes of patients treated with and without adjunctive rifampin for diabetic foot osteomyelitis?

**Findings:**

In this cohort study that analyzed 6174 veterans with diabetic foot osteomyelitis, a significantly lower rate of a combined amputation or mortality end point was observed in those treated with rifampin (26.9%) compared with those without rifampin (37.0%).

**Meaning:**

Based on these findings, adjunctive rifampin may be a useful antimicrobial addition in the treatment of diabetic foot osteomyelitis, and a rigorous evaluation of its effect is warranted.

## Teaser

This cohort study compares amputation and mortality rates among patients treated in the Veterans Health Administration for diabetic foot osteomyelitis with and without rifampin.

## Introduction

Amputation is a serious and unfortunate consequence of lower limb osteomyelitis in patients with diabetes. Fifteen percent of patients with diabetes experience a foot ulcer. Depending on the severity and chronicity of infection, 20% to 60% of diabetic foot infections (DFI) involve bone.^1^ When a foot infection in a patient with diabetes extends to bone (diabetic foot osteomyelitis [DFO]), a 4-fold increased risk of amputation is present.^2^

Antimicrobial therapy for DFI varies widely. A study of treatment regimens provided throughout the Veterans Health Administration (VHA) for DFI found that 17 different regimens were provided to 1% or more of such patients.^3^ The limited available comparative efficacy data preclude identification of a preferred regimen for these infections.

Rifampin has unique antimicrobial properties that make it a useful adjunctive therapy for osteomyelitis. Rifampin penetrates osteoblasts and retains antimicrobial activity within the cells.^4^ Biofilms in osteomyelitis also contribute to the therapeutic challenge of osteomyelitis.^5^ Bacteria in biofilms respond poorly to standard antimicrobial therapy. Rifampin penetrates biofilms and retains activity within them. Although metabolically active bacteria are readily killed by appropriately targeted antimicrobials, some bacteria within biofilms enter a dormant state and are termed *persister cells*.^6^ When antimicrobial therapies are withdrawn, the persister cells may reactivate to cause recurrent infections. Rifampin also demonstrates potent activity against persister cells in biofilms that exceeds that of any other currently available antibiotic.^7^

Based on a systemic review of studies of bone penetration and pharmacokinetics, animal models, multiple retrospective studies, and several prospective, randomized clinical trials, adjunctive rifampin has been described to be beneficial for treating osteomyelitis outside the context of DFI.^8^ Intravenous therapy for severe DFI is selected most often in the United States, and rifampin is not commonly used in North America to treat osteomyelitis. In contrast, oral therapy including rifampin is commonly prescribed in Europe.^9,10^ In a recent trial conducted in the United Kingdom examining oral vs intravenous therapy for bone and joint infections,^11^ open-label adjunctive oral rifampin was prescribed for 48% of participants. Although outcomes of those treated with and without rifampin did not differ significantly, the initiation and duration of rifampin treatment was highly variable, and DFO represented a small subset of the treatment population, with foot infections representing only 20% of cases and diabetes present in 20% of all study participants.^11^ In retrospective and prospective randomized clinical trials, the addition of rifampin has reduced relapse rates from chronic osteomyelitis, improving arrest of osteomyelitis by 28% to 42% compared with regimens without rifampin.^12,13,14^ The effect of adjunctive rifampin in DFO remains an open question with little direct assessment in previous research.

The safety profile of rifampin is similar to that of other agents used to treat staphylococcal osteomyelitis.^15^ In contrast, drug interactions with medications metabolized by the cytochrome P450 system, including warfarin and some antidiabetic agents, pose a barrier to the use of rifampin. The range of observed antibiotic treatments, the high burden of comorbidities, and differences among nonpharmacological therapies offered by podiatry and vascular surgery specialists present challenges to assessing the effectiveness of rifampin in treating DFO.

The VHA’s Cooperative Studies Program is currently enrolling patients in a multisite randomized clinical trial, Investigation of Rifampin to Reduce Pedal Amputations for Osteomyelitis in Diabetics (VA INTREPID), testing the effect of adjunctive rifampin in the treatment of DFO.^16^ In preparation for that trial, we analyzed the use of rifampin in a historic cohort of veterans with DFO to characterize the use of adjunctive rifampin and assess its association with subsequent amputations and deaths. In this observational cohort and in the ongoing randomized clinical trial,^16^ amputation-free survival was selected as an end point that is patient centered and unambiguous. Previous studies of populations with DFO^17,18^ have considered a time-to-healing end point and observed similar or shorter healing times in patients undergoing amputation. However, clinical experience and research on patient attitudes show that amputation is more feared than death in a population with diabetic foot disease.^19^ Thus, our choice of end point was guided by patient attitudes, clinical relevance, and ease of ascertainment.

## Methods

Study approval was obtained from the VA Northeast Ohio Healthcare System institutional review board, which waived the need for informed consent for the use of VHA data, given minimal risk of the study, and that the waiver would not adversely affect the rights and welfare of the participants. This study followed the Strengthening the Reporting of Observational Studies in Epidemiology (STROBE) reporting guideline.

Using Corporate Data Warehouse tables accessed through the Veterans Affairs Informatics and Computing Infrastructure, VHA patients with diabetes and osteomyelitis of the foot or ankle (*International Classification of Diseases, Ninth Revision* [*ICD-9*], codes 730.07, 730.17, and 730.27) from January 1, 2009, through December 31, 2013, were identified. Patients with additional diagnoses (*ICD-9* codes 682.6, 707.13, 715.98, 730.07, 730.28, and 824.8) consistent with ankle involvement or bone infections at other sites were excluded to limit the analysis to patients with DFO. To focus on antibiotic treatment regimens with and without rifampin, the population was further restricted to patients alive at 90 days after diagnosis without existing major amputation (ankle or above) and who received VHA-dispensed antibiotics within 6 weeks of diagnosis. Amputations below the ankle that occurred within 90 days of diagnosis were not regarded as events, and these patients were considered to remain at risk and included in the analysis. Amputations and deaths occurring from 90 days to 2 years after the index diagnosis date were considered events of interest. Rifampin is uncommonly used in the treatment of DFO in the VHA and was not dispensed for DFO in most of the VHA facilities where cases were diagnosed or treated; hence, patients from these facilities (50% of the initial DFO cohort) were excluded from analysis to minimize facility bias.

Patients may receive variable durations of antibiotic therapy after diagnosis of DFO (eg, while awaiting debridement of necrotic tissue or completion of revascularization procedures), and rifampin may be prescribed at various points of disease progression (selected early with intention to cure, added to a failed regimen after a further debridement and evaluation, or prescribed at a remote date and unrelated to the DFO diagnosis). Therefore, we attempted to identify patients with early initiation of rifampin therapy and with rifampin exposure of sufficient duration to meaningfully affect outcomes. Patients initiating rifampin therapy within 6 weeks of the index osteomyelitis diagnosis and receiving the drug for at least 14 days within 90 days after diagnosis were considered to have been treated with rifampin. Patients who did not receive any rifampin within 90 days after diagnosis were considered not treated with rifampin and served as the comparator group to rifampin-treated patients. Patients receiving fewer than 14 days of rifampin therapy or starting rifampin therapy more than 6 weeks after diagnosis were excluded from the analysis. The 6-week initiation and 90-day treatment windows were chosen to identify cases of presumptive rifampin use with intention to cure DFO, allowing for possible stops and starts for debridement or other procedures while excluding other uses. The 14-day treatment period was chosen to ensure clinically meaningful rifampin therapy.

For patients in the analysis data set, demographics, comorbidities, clinical and microbiological laboratory findings, debridement procedures, infectious disease consultations, and medications dispensed during inpatient stays or through VHA outpatient pharmacies were extracted from the Corporate Data Warehouse linked to this health care system. Characteristics of patients, infections, and treatments were compared between cases treated with and without rifampin. Microbiological specimens collected within 2 weeks before or 6 weeks after DFO diagnosis with a topography consistent with DFO (bone, blood, foot, ulcer, or tissue) were reviewed for all patients in the data set, and those with *Staphylococcus aureus* recorded in microbiological laboratory results were identified as positive for *S aureus*. In patients without specimens collected or without *S aureus* detected in specimens, *S aureus* was considered not identified. Outpatient pharmacy fills in the 6 weeks before diagnosis were queried for the following commonly prescribed drugs with major interactions with rifampin as defined by Micromedex: opioids, warfarin, phenytoin sodium, quetiapine fumarate, and aripiprazole. Patients with a warfarin prescription, the most frequently observed of these drugs, were coded as such. Insulin fills in the 6 months before diagnosis were identified to characterize diabetes treatment. Hemoglobin A_1c_ and creatinine levels in the year before diagnosis were queried, and the value closest in time to the date of diagnosis was retained.

Differences in mean values of continuous variables were compared using 2-tailed independent-samples *t* tests. Differences in binary variables and event rates of amputation and death in the 2 years after the index diagnosis date were compared using χ^2^ tests or Fisher exact tests in instances of small cell counts. A combined time-to-event end point of mortality or amputation was analyzed using Kaplan-Meier survival curves and a log-rank test to compare treatment groups. Using logistic regression, we estimated the odds of the combined end point among patients treated with rifampin relative to patients treated without rifampin in unadjusted and adjusted models. We assessed the possible interaction of rifampin and *S aureus* in the subset of patients with microbiological cultures. For patients in whom *S aureus* was isolated, we summarized available susceptibility data and performed a subgroup analysis.

Antibiotic combinations were summarized for patients treated with rifampin. Agents were considered to be prescribed in combination with rifampin when 3 or more days of overlap with rifampin treatment was observed. Manual review of clinical notes, laboratory results, and dispensed medications in the electronic health record for 20 rifampin-treated cases was performed to check diagnoses and treatment regimens and to characterize database limitations specific to this cohort. Corporate Data Warehouse queries were performed using SQL Server 2014 Management Studio; extracted data were summarized and analyzed in R, version 3.5.1 (R Project for Statistical Computing), using the survival package for time-to-event analyses. Two-sided *P* < .05 indicated statistical significance.

## Results

**Figure 1.**
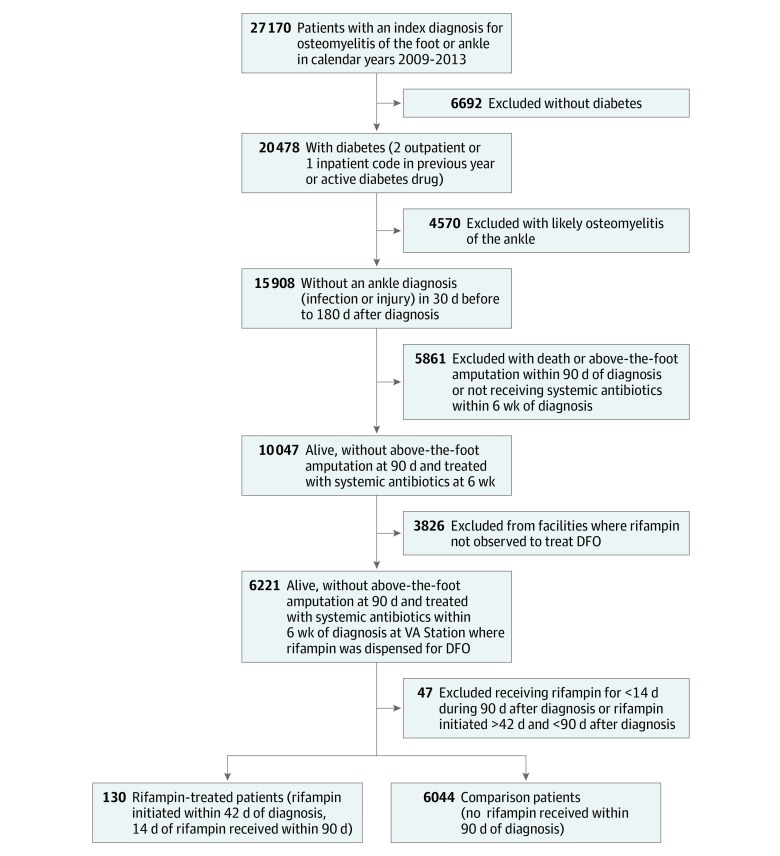
Flow Diagram of Inclusion and Exclusion Criteria DFO indicates diabetic foot osteomyelitis; VA, Department of Veterans Affairs.

Of 27 170 nationwide VHA patients with an *ICD-9* diagnostic code for foot or ankle osteomyelitis, 15 908 met DFO criteria (presence of diabetes and absence of ankle involvement, based on *ICD-9* codes). Exclusions of deaths and major amputation within 90 days of diagnosis and patients not treated with systemic antibiotics dispensed by VHA within 6 weeks of diagnosis left 10 047 patients, of whom 6221 were treated at facilities where rifampin was dispensed for DFO. After removing patients receiving late and/or short durations of rifampin, the remaining analysis population was composed of 130 patients treated with rifampin and 6044 treated without rifampin, for a total study population of 6174 patients (6085 men [98.6%] and 89 women [1.4%]; mean [SD] age, 64.9 [9.7] years) (Figure 1).

Several statistically significant differences were observed between rifampin treatment and comparison groups. Patients treated with rifampin were younger (mean [SD] age, 62.2 [9.4] vs 64.9 [9.6] years; *P* = .001), had fewer comorbidities (mean [SD] Charlson comorbidity index score, 3.5 [1.8] vs 4.0 [2.2]; *P* = .006), more often had a consultation with infectious diseases specialists (63 of 130 [48.5%] vs 1960 of 6044 [32.4%]; *P* < .001), and more frequently had *S aureus* identified in cultures (55 of 130 [42.3%] vs 1755 of 6044 [29.0%]; *P* = .001) than comparison patients (Table 1). A wide range of agents and antibiotic classes was prescribed with rifampin to treat DFO. Rifampin was most frequently observed in combination with fluoroquinolones (62 [47.7%]), tetracyclines (37 [28.5%]), sulfa or trimethoprim (27 [20.8%]), and vancomycin (20 [15.4%]).

**Table 1.** Patient Characteristics and Clinical Outcomes by Treatment Group

Patient Variable	Treatment Group^a^		*P* Value^b^
	Comparator (n = 6044)	Rifampin (n = 130)	
Patient demographic characteristics			
Male, No. (%)	5955 (98.5)	130 (100)	.27
Age, mean (SD), y	64.9 (9.6)	62.2 (9.4)	.001
Race, No. (%)			
White	4266 (70.6)	96 (73.8)	.59
Black	1345 (22.3)	24 (18.5)	
Other or missing	433 (7.2)	10 (7.7)	
Ethnicity, No. (%)			
Non-Latino	5353 (88.6)	117 (90.0)	.84
Latino	469 (7.8)	8 (6.2)	
Other or missing	222 (3.7)	5 (3.8)	
Patient comorbidities			
Charlson comorbidity index score, mean (SD)	4.0 (2.2)	3.5 (1.8)	.006
Heart conditions, No. (%)	1689 (27.9)	26 (20.0)	.06
Peripheral vascular disease, No. (%)	2281 (37.7)	40 (30.8)	.13
Stroke, No. (%)	1103 (18.2)	18 (13.8)	.24
Pulmonary disease, No. (%)	1258 (20.8)	29 (22.3)	.76
Liver disease, No. (%)	517 (8.6)	9 (6.9)	.62
Renal disease, No. (%)	1823 (30.2)	36 (27.7)	.61
Patient laboratory results before DFO			
No HbA_1c_ level in prior year, No. (%)	399 (6.6)	11 (8.5)	.65
HbA_1c_ level, No. (%)			
<7.5%	2454 (40.6)	48 (36.9)	
7.5%-9.4%	1895 (31.4)	45 (34.6)	
≥9.5%	1296 (21.4)	26 (20.0)	
No creatinine level in prior year, No. (%)	399 (6.6)	10 (7.7)	.89
Creatinine level, No. (%)			
<1.2 mg/dL	2694 (44.6)	56 (43.1)	
1.2-4.9 mg/dL	2742 (45.4)	59 (45.4)	
≥5.0 mg/dL	209 (3.5)	5 (3.8)	
Patient pharmacy fills, No. (%)			
Insulin (previous 6 mo)	3316 (54.9)	66 (50.8)	.40
Warfarin (previous 6 wk)	605 (10.0)	9 (6.9)	.31
Infection and treatment, No. (%)			
Infectious disease consultation	1960 (32.4)	63 (48.5)	<.001
Debridement	3094 (51.2)	68 (52.3)	.87
Microbiological cultures	4747 (78.5)	99 (76.2)	.58
*Staphylococcus aureus* identified	1755 (29.0)	55 (42.3)	.001
Clinical outcomes at 2 y, unadjusted			
Amputation	1390 (23.0)	23 (17.7)	.19
Death	1056 (17.5)	14 (10.8)	.06
Amputation or death	2250 (37.2)	35 (26.9)	.02

Abbreviations: DFO, diabetic foot osteomyelitis; HbA_1c_, hemoglobin A_1c_.

SI conversion factors: to convert creatinine to micromoles per liter, multiply by 88.4; HbA_1c_ to proportion of total hemoglobin, multiply by 0.01.

^a^ Percentages have been rounded and may not total 100.

^b^ Continuous variables are compared using a 2-sample *t* test; categorical variables, with a χ^2^ test or Fisher exact test; unadjusted *P* values presented.

**Figure 2.**
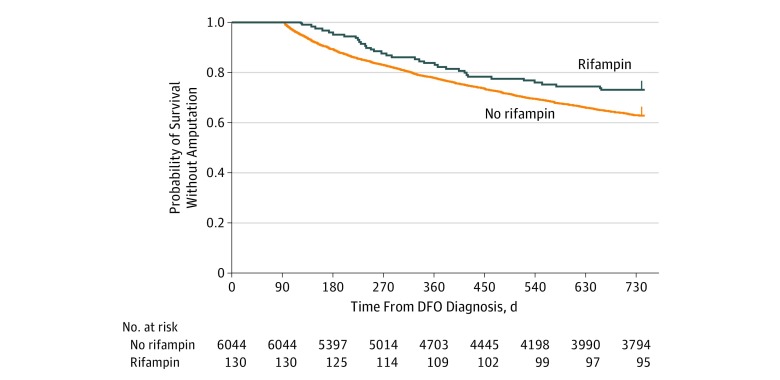
Kaplan-Meier Curves of Treatment Groups in Analysis Population DFO indicates diabetic foot osteomyelitis.

In the 2 years after DFO diagnosis, lower rates of amputation and death were observed among patients receiving rifampin (Table 1). Using a log-rank test to compare treatment groups, amputation-free survival was significantly higher in patients treated with rifampin (amputation or death recorded for 35 of 130 patients [26.9%] vs 2250 of 6044 patients [37.2%]; *P* = .02) (Figure 2). Using a multivariable logistic regression to estimate the odds of events while adjusting for patient, infection, and treatment variables, we observed a significant association of rifampin with lower odds of events (OR, 0.65; 95% CI, 0.43-0.96; *P* = .04) (model results in Table 2).

**Table 2.** Bivariate and Multivariable Logistic Regression Estimating Amputation or Death in 2 Years

Model Variables	OR (95% CI)		Multivariable *P* Value
	Bivariate Logistic	Multivariable Logistic	
Age at diagnosis, y	1.02 (1.01-1.02)	1.01 (1.01-1.02)	<.001
Charlson comorbidity index score, continuous	1.15 (1.12-1.18)	1.14 (1.11-1.17)	<.001
Race			
Black vs white	1.00 (0.88-1.13)	0.94 (0.82-1.07)	.33
Other/missing vs white	0.91 (0.74-1.11)	0.91 (0.72-1.14)	.41
Ethnicity			
Latino vs non-Latino	0.97 (0.80-1.17)	0.94 (0.77-1.16)	.58
Other/missing vs non-Latino	0.97 (0.74-1.28)	1.07 (0.78-1.45)	.66
Hemoglobin A_1c_ level, %			
7.5-9.4 vs <7.5	1.07 (0.95-1.21)	1.17 (1.03-1.33)	.02
≥9.5 vs <7.5	1.02 (0.89-1.17)	1.22 (1.05-1.42)	.009
Not measured vs <7.5	1.26 (1.02-1.56)	1.54 (1.23-1.92)	<.001
Creatinine level, mg/dL			
1.2-4.9 vs <1.2	1.22 (1.09-1.36)	1.01 (0.90-1.14)	.84
≥5.0 vs <1.2	2.39 (1.81-3.17)	1.84 (1.37-2.47)	<.001
Not measured vs <1.2	1.15 (0.92-1.42)	1.03 (0.82-1.30)	.77
Insulin fill (6 mo before DFO)	1.11 (1.00-1.23)	1.02 (0.91-1.14)	.77
Warfarin fill (6 wk before DFO)	1.18 (1.00-1.40)	1.00 (0.83-1.19)	.96
Infectious disease consultation	1.19 (1.07-1.33)	1.13 (1.01-1.27)	.03
Debridement	1.05 (0.95-1.16)	1.01 (0.91-1.13)	.82
Any microbiological cultures	1.28 (1.13-1.46)	1.25 (1.08-1.44)	.002
Any isolated *Staphylococcus aureus*	1.05 (0.94-1.18)	0.99 (0.88-1.12)	.86
Treated with rifampin	0.62 (0.42-0.91)	0.65 (0.43-0.96)	.04

Abbreviations: DFO, diabetic foot osteomyelitis; OR, odds ratio.

In addition to the associations of age and comorbidity with our combined end point, we observed significant associations of hemoglobin A_1c_ and creatinine (OR for ≥5.0 vs <1.2 mg/dL, 1.84; 95% CI, 1.37-2.47; *P* < .001) levels with amputation-free survival, suggesting the potential roles of glycemic control and renal function on outcomes for patient with DFO. An additional model was estimated using the subset of patients with microbiological cultures, testing the interaction of rifampin and identified *S aureus*. No interaction was detected in the subset of 4846 patients.

We analyzed a microbiological subgroup of patients with *S aureus* infection in whom we found similar rates of methicillin-resistant *S aureus* in patients treated with and without rifampin (of 1734 with susceptibility results, 24 of 49 [49.0%] vs 721 of 1685 [42.8%]). Consistent with the absence of an interaction in our multivariable model, we found the event rates by treatment group in the *S aureus* subset matched those in our full analysis cohort (15 of 55 [27.3%] in those treated with rifampin vs 669 of 1755 [38.1%] in those treated without rifampin). Among the 1277 *S aureus* isolates identified with available susceptibility results for rifampin, 1271 of 1277 (99.5%) were rifampin susceptible.

## Discussion

In this analysis, we observed that patients treated with rifampin experienced lower rates of death and amputation than patients not treated with rifampin, although in the presence of notable confounders. In this cohort, patients receiving rifampin were significantly younger, had fewer comorbidities, had greater infectious diseases specialty involvement, and were more often infected with *S aureus* than patients not receiving rifampin. Given the drug-drug interactions of medications commonly prescribed with rifampin and the adverse effect profile of this drug, a younger population with fewer comorbidities receiving rifampin is not surprising. These results may also indicate that in a comparatively younger and healthier population with diabetes, more aggressive antibiotic therapy is provided in attempts to avoid amputations.

### Limitations

Limitations of the analysis included the use of administrative data to find cases through diagnosis codes, the varied backbone antibiotic regimens, the inability to gather data on antibiotics dispensed through contracted non-VHA services, and unaccounted-for variations in nonpharmacological interventions, including wound care, off-loading methods, and procedures to enhance vascular perfusion. In addition, other important clinical factors such as access to specialized care and frequency of follow-up visits were not considered.

We controlled for identified confounders and additional covariates in a multivariable logistic regression model and found a persistent association of rifampin with amputation-free survival, but we acknowledge that unaddressed confounding effects of these variables and unmeasured confounders may exist. Identifying, extracting, and balancing all such variables to estimate a causal effect of adjunctive rifampin were beyond the scope of this study. Our definition of rifampin treatment excluded patients who received short courses of rifampin treatment (<14 days) or in whom rifampin was initiated more than 6 weeks after diagnosis. These early discontinuations and late starts might represent distinct patient populations of interest when considering management of adverse effects and use of rifampin as salvage therapy, respectively, but fell outside of our analysis population. In addition, by excluding deaths and major amputations occurring in the first 90 days following diagnosis, we introduced survivorship bias to our analysis to define distinct windows for treatment and risk.

## Conclusions

Results from this observational cohort study, coupled with existing evidence from small clinical trials, suggest that adjunctive rifampin therapy may be a useful antimicrobial strategy in the treatment of DFO. A rigorous evaluation of its effects with a large interventional study is warranted.

## References

[1] Lipsky BA, Berendt AR, Cornia PB, ; Infectious Diseases Society of America 2012 Infectious Diseases Society of America clinical practice guideline for the diagnosis and treatment of diabetic foot infections. Clin Infect Dis. 2012;54(12):-. doi:10.1093/cid/cis346 22619242

[2] Mutluoglu M, Sivrioglu AK, Eroglu M, The implications of the presence of osteomyelitis on outcomes of infected diabetic foot wounds. Scand J Infect Dis. 2013;45(7):497-503. doi:10.3109/00365548.2013.765589 23384323

[3] Fincke BG, Miller DR, Christiansen CL, Turpin RS Variation in antibiotic treatment for diabetic patients with serious foot infections: a retrospective observational study. BMC Health Serv Res. 2010;10:193. doi:10.1186/1472-6963-10-193 20604922 PMC2914722

[4] Valour F, Trouillet-Assant S, Riffard N, Antimicrobial activity against intraosteoblastic *Staphylococcus aureus*. Antimicrob Agents Chemother. 2015;59(4):2029-2036. doi:10.1128/AAC.04359-14 25605365 PMC4356812

[5] Conlon BP, Rowe SE, Lewis K Persister cells in biofilm associated infections. Adv Exp Med Biol. 2015;831:1-9. doi:10.1007/978-3-319-09782-4_1 25384659

[6] Lebeaux D, Chauhan A, Rendueles O, Beloin C From in vitro to in vivo models of bacterial biofilm-related infections. Pathogens. 2013;2(2):288-356. doi:10.3390/pathogens2020288 25437038 PMC4235718

[7] Conlon BP, Nakayasu ES, Fleck LE, Activated ClpP kills persisters and eradicates a chronic biofilm infection. Nature. 2013;503(7476):365-370. doi:10.1038/nature12790 24226776 PMC4031760

[8] Spellberg B, Lipsky BA Systemic antibiotic therapy for chronic osteomyelitis in adults. Clin Infect Dis. 2012;54(3):393-407. doi:10.1093/cid/cir842 22157324 PMC3491855

[9] Senneville E, Nguyen S Current pharmacotherapy options for osteomyelitis: convergences, divergences and lessons to be drawn [review]. Expert Opin Pharmacother. 2013;14(6):723-734. doi:10.1517/14656566.2013.780596 23496344

[10] Senneville E, Lombart A, Beltrand E, Outcome of diabetic foot osteomyelitis treated nonsurgically: a retrospective cohort study. Diabetes Care. 2008;31(4):637-642. doi:10.2337/dc07-1744 18184898

[11] Li HK, Rombach I, Zambellas R, ; OVIVA Trial Collaborators Oral versus intravenous antibiotics for bone and joint infection. N Engl J Med. 2019;380(5):425-436. doi:10.1056/NEJMoa1710926 30699315 PMC6522347

[12] Van der Auwera P, Klastersky J, Thys JP, Meunier-Carpentier F, Legrand JC Double-blind, placebo-controlled study of oxacillin combined with rifampin in the treatment of staphylococcal infections. Antimicrob Agents Chemother. 1985;28(4):467-472. doi:10.1128/AAC.28.4.467 3907494 PMC180285

[13] Norden CW, Bryant R, Palmer D, Montgomerie JZ, Wheat J Chronic osteomyelitis caused by *Staphylococcus aureus*: controlled clinical trial of nafcillin therapy and nafcillin-rifampin therapy. South Med J. 1986;79(8):947-951. doi:10.1097/00007611-198608000-00008 3526570

[14] Zimmerli W, Widmer AF, Blatter M, Frei R, Ochsner PE; Foreign-Body Infection (FBI) Study Group Role of rifampin for treatment of orthopedic implant-related staphylococcal infections: a randomized controlled trial. JAMA. 1998;279(19):1537-1541. doi:10.1001/jama.279.19.1537 9605897

[15] Valour F, Karsenty J, Bouaziz A, ; Lyon BJI Study Group Antimicrobial-related severe adverse events during treatment of bone and joint infection due to methicillin-susceptible *Staphylococcus aureus*. Antimicrob Agents Chemother. 2014;58(2):746-755. doi:10.1128/AAC.02032-13 24247130 PMC3910824

[16] ClinicalTrials.gov Investigation of Rifampin to Reduce Pedal Amputations for Osteomyelitis in Diabetics (VA INTREPID). NCT03012529. February 23, 2018 https://clinicaltrials.gov/ct2/show/NCT03012529. Accessed February 23, 2018.

[17] Arias M, Hassan-Reshat S, Newsholme W Retrospective analysis of diabetic foot osteomyelitis management and outcome at a tertiary care hospital in the UK. PLoS One. 2019;14(5):e0216701. doi:10.1371/journal.pone.0216701 31095593 PMC6522026

[18] Lázaro-Martínez JL, Aragón-Sánchez J, García-Morales E Antibiotics versus conservative surgery for treating diabetic foot osteomyelitis: a randomized comparative trial. Diabetes Care. 2014;37(3):789-795. doi:10.2337/dc13-1526 24130347

[19] Wukich DK, Raspovic KM, Suder NC Patients with diabetic foot disease fear major lower-extremity amputation more than death. Foot Ankle Spec. 2018;11(1):17-21. doi:10.1177/1938640017694722 28817962

